# Poly(Lactic Acid) Filled with Cassava Starch-g-Soybean Oil Maleate

**DOI:** 10.1155/2013/860487

**Published:** 2013-11-05

**Authors:** Nopparut Kiangkitiwan, Kawee Srikulkit

**Affiliations:** ^1^Department of Materials Science, Faculty of Science, Chulalongkorn University, Bangkok 10330, Thailand; ^2^Center of Excellence on Petrochemicals and Materials Technology, Chulalongkorn University, Bangkok 10330, Thailand

## Abstract

Poly(lactic acid), PLA, is a biodegradable polymer, but its applications are limited by its high cost and relatively poorer properties when compared to petroleum-based plastics. The addition of starch powder into PLA is one of the most promising efforts because starch is an abundant and cheap biopolymer. However, the challenge is the major problem associated with poor interfacial adhesion between the hydrophilic starch granules and the hydrophobic PLA, leading to poorer mechanical properties. In this paper, soybean oil maleate (SOMA) was synthesized by grafting soybean oil with various weight percents of maleic anhydride (MA) using dicumyl peroxide (DCP) as an initiator. Then, SOMA was employed for the surface modifying of cassava starch powder, resulting in SOMA-g-STARCH. The obtained SOMA-g-STARCH was mixed with PLA in various weight ratios using twin-screw extruder, resulting in PLA/SOMA-g-STARCH. Finally, the obtained PLA/SOMA-g-STARCH composites were prepared by a compression molding machines. The compatibility, thermal properties, morphology properties, and mechanical properties were characterized and evaluated. The results showed that the compatibility, surface appearance, and mechanical properties at 90 : 10 and 80 : 20 ratios of PLA/SOMA-g-STARCH were the best.

## 1. Introduction 

The petroleum-derived plastics have been used extensively, causing tons of thousands of plastic waste. The improper disposal of petroleum-derived plastics leads to environmental pollution which has aroused much interest in searching for substitutes [1]. Recently, biodegradable and renewable polymers have been increasingly developed. Generally, polymers from renewable resources can be classified into three groups: (1) natural polymers such as starch, protein, and cellulose; (2) synthetic polymers from natural monomers such as polybutylene succinate (PBS) and polylactide (PLA); and (3) polymers from microbial fermentation such as polyhydroxybutyrate [2]. These polymers are aliphatic polyesters which are biodegradable and compostable thermoplastics derived from renewable resources, such as starch and sugar cane [1]. PLA and PBS are the most promising thermoplastic polymers in this regard. They are compostable which is perfectly suitable for consumer goods and packaging applications. They are derived from renewable resources such as corn, sugar cane and cassava. They are biodegradable polymers which are nontoxical and acceptable mechanical performance but their applications, particularly PLA, are limited by the high cost and relatively poorer properties when compared to petroleum-based plastics. PBS is semicrystalline polymer which exhibits the flexibility when compared to PLA. On the other hand, PLA is brittle polymer due to its high *T*_*g*_, low crystallinity and low thermal stability. With respect to its chemistry, PLA is synthesized by direct condensation polymerization of the lactic acid monomers or by ring opening polymerization of lactide monomer. There have been several possibilities to modify its property such as the nucleating agent addition, polymer composites, and polymer blend preparation with natural fillers such as starch or cellulose and with natural rubber, respectively [3–6]. 

 The addition of starch into PLA is one of the most promising efforts due its abundant and cheap biopolymer particularly cassava starch since it is the fifth most abundant starch crop produced in the world and the third most important food source for inhabitants of tropical regions. As a result, starch has been used as filler for environmentally friendly plastics [7]. However, the challenge is the major problem associated with poor interfacial adhesion between the hydrophilic starch granules and the hydrophobic PLA, leading to poorer mechanical properties. Therefore, several strategies have been adopted to improve the compatibility such as by using compatibilizers or reactive coupling agents [1, 8, 9]. Especially, maleic anhydride- (MA) based compatibilizing agents are the most popular due to their good chemical reactivity, low toxicity, and low potential to polymerize itself under free radical grafting conditions [5]. Wang et al. prepared thermoplastic dry starch (DTPS)/PLA blends by using MA as compatibilizer and dicumyl peroxide (DCP) as initiator to enhance the compatibility between DTPS and PLA [1]. Glycerol was employed as a plasticizer for dry starch to avoid the depolymerization of hydrophobic PLA during melt processing. The plasticization of starch and its compatibility modification with PLA was accomplished in a single-screw extruder by one-step reactive extrusion. The results have been reported that the physical properties and compatibility of DTPS/PLA blends were improved. 

 This research was focused on the effect of soybean oil maleate grafted cassava starch as a filler on the properties improvement of PLA. The soybean oil maleate grafted cassava starch was easily synthesized by the maleation of soybean oil with maleic anhydride [10]. Thus, obtained soybean oil maleate grafted cassava starch was mixed with PLA by twin-screw extruder using various weight ratios. The compatibility was evaluated and discussed in detail. 

## 2. Materials and Methods

### 2.1. Materials

PLA pellet (2003D grade) by NatureWorks was purchased from BC Polymers Marketing Co., Ltd. (Bangkok, Thailand). It is transparent polymer with a glass transition temperature (*T*_*g*_) of 58–60°C and a density of 1.24 g/cm^3^, as reported by the manufacturer. Cassava starch flour was purchased from Thai Wah Food Products Public Company Limited (Bangkok, Thailand). Soybean oil was supplied from Siam Chemical Industry (Thailand). Maleic anhydride (≥98% purity) produced by Sigma Aldrich, dicumyl peroxide (98% purity) manufactured by Sigma Aldrich, and acetone (AR grade) were purchased from the local distributor (FACOBIS Co., Ltd.).

### 2.2. Preparation of Soybean Oil Maleate

Soybean oil (100 g) was charged into a 500 mL glass reactor. Then, 10 g or 20 g of MA powder (two reaction batches; batch one: MA 10 wt% based on soybean oil and batch two: MA 20 wt% based on soybean oil) was added and continuously stirred. Following that, DCP (3 wt% of MA) was used as a free radical initiator. The reaction was allowed to continue at temperature of 170°C for 2 hrs. The viscous yellowish liquid of soybean oil maleate (SOMA) was obtained. SOMA10 and SOMA20 were assigned to soybean oil maleate having anhydride content of 10 wt% and 20 wt%, respectively. 

### 2.3. Preparation of Cassava Starch-g-Soybean Oil Maleate

50 g SOMA and 50 g starch powder at weight ratio of 1 : 1 were well mixed using a mechanical stirrer. After that, the mixture was put in the hot-air oven and the temperature was set at 120°C for 2 hours. It was anticipated that at this condition, SOMA underwent the ring opening reaction with starch hydroxyl group, yielding SOMA-g-STARCH. Then, the crude product was washed with acetone to remove unreacted SOMA. Two sorts of SOMA-g-STARCH were prepared which were assigned to SOMA10-g-STARCH and SOMA20-g-STARCH for SOMA-g-STARCHs prepared from SOMA10 and SOMA20, respectively. The obtained SOMA-g-STARCHs were dried in an oven at 50°C and ground prior to subsequent experiment. 

### 2.4. Preparation of PLA Filled with Cassava Starch-g-Soybean Oil Maleate

The obtained SOMA-g-STARCH (SOMA10-g-STRACH and SOMA20-g-STRACH) was mixed with PLA in various PLA : SOMA-g-STARCH weight ratios of 90 : 10, 80 : 20, 70 : 30, 60 : 40, and 50 : 50 using twin-screw extruder and the barrel zone temperature was set at 135, 160, 170, 180, and 180°C fore zone 1, zone 2, zone 3, and zone 4, respectively.

### 2.5. Characterization and Testing

Fourier transform infrared spectroscopy was conducted using Nicolet FTIR spectrometer (Nicolet 6700) recorded in the range of 3500–1000 cm^−1^. Proton NMR was recorded on Varian ^UNITY^INOVA 500 Hz spectrometer using deuterated acetone as a medium. Morphology was observed by a JEOL, JSM-6480LV scanning electron microscope (Tokyo, Japan) using acceleration voltage of 22 kV. Colored Appearance was evaluated by dyeing 1 wt% red disperse dye using H24 Newave Lab Equipment (Taiwan). TGA analysis was conducted on Mettler Toledo TGA/SDTA851^e^ thermalgravimetric analyzer (Columbus, Ohio) which was carried out under nitrogen atmosphere with the flow rate of 20 mL/min and heating from 25°C to 600°C at heating rate of 10°C/min. Impact strength was determined by izod impact tester (GOTECH) according to ASTM D256 standard, and tensile properties were determined by universal testing machine (LR 100 K) according to ASTM D638 standard and using load cell of 100 kN and crosshead speed at 50 mm/min.

## 3. Results and Discussion

### 3.1. Characterization of Soybean Oil Maleate

**Scheme 1:**
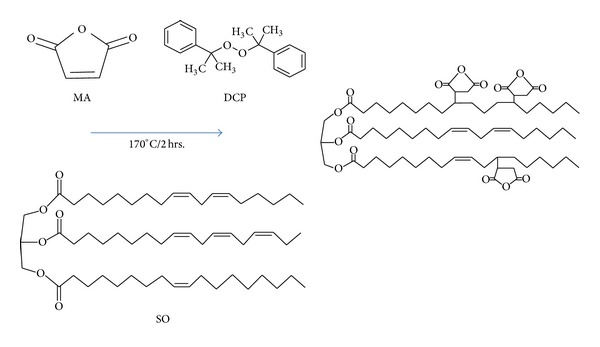
Representative grafting reaction of soybean oil with maleic anhydride using dicumyl peroxide as an initiator.

**Figure 1:**
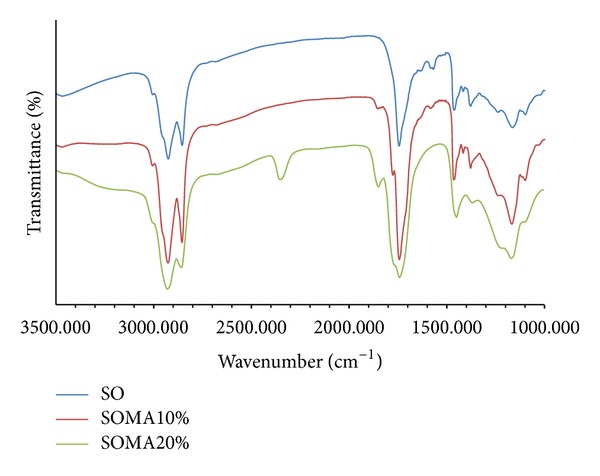
FTIR spectra of soybean oil and soybean oil maleate.

The graft reaction between soybean oil and maleic anhydride initiated by DCP to produce SOMA is proposed by Scheme 1. The appearance of SOMA is viscous and brownish. Upon standing for several days, the unreacted MA is crystallized and discarded, leaving only SOMA liquid. The SOMA functional groups were identified by FTIR analysis of which FTIR spectra are presented in Figure 1. The typical absorption band of carbonyl ester group of soybean oil is found to be 1720 cm^−1^. From the spectra of SOMA10 and SOMA20, the additional absorption bands at 1775 cm^−1^ and 1850 cm^−1^ are observed, corresponding to the symmetric and asymmetric stretching of C=O in the pendent anhydride group. Their absorption intensities tend to increase with an increase in the amount of applied MA, indicating that the more the amount of added MA the more the amount of anhydride ring content. This anhydride ring remains intact since no absorption peaks related to –OH stretching in the region of 3,500 cm^−1^ due to the opening of the anhydride were observed [6]. The presence of anhydride ring is important for the subsequent grafting reaction.

**Figure 2:**
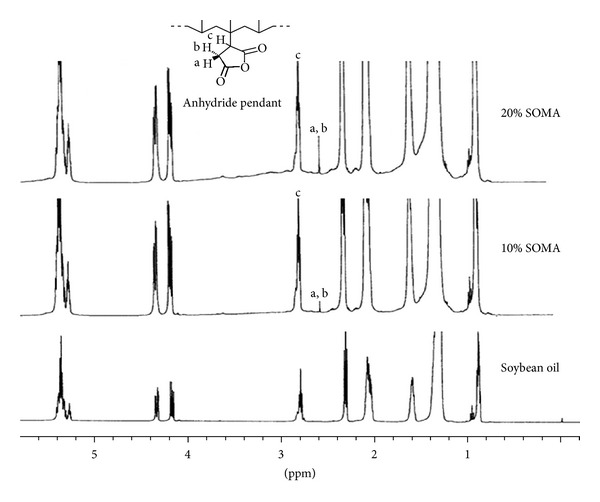
^1^H NMR spectra of soybean oil and soybean oil maleate.

The ^1^H-NMR technique was employed to confirm the structure of SOMA.  Figure 2 shows the NMR spectra of SOMA and SO. The signals of methylene protons of anhydride pendant are obviously seen at around 2.60–2.70 ppm (a, b) and at around 2.80–2.90 ppm (c) which are not present in the spectrum of SO [7]. Based on two techniques, it is certain that SOMA was successfully synthesized and the possible structure is proposed as shown in Scheme 1. 

### 3.2. Cassava Starch-g-Soybean Oil Maleate (SOMA-g-STARCH)

**Figure 3:**
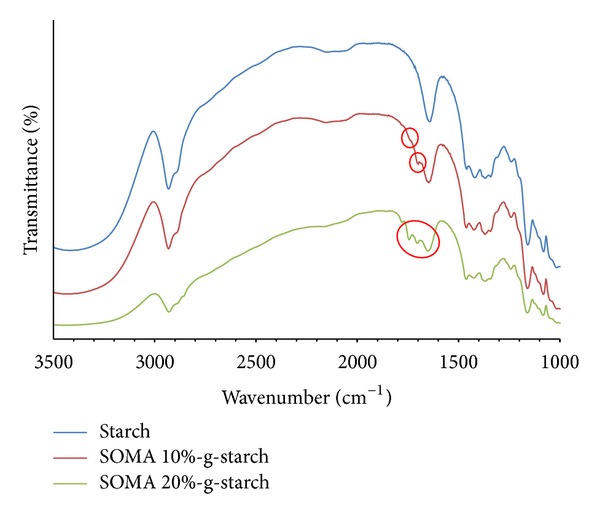
FTIR spectra of starch and soybean oil-g-cassava starch powder.

SOMA-g-STARCH represents surface modified starch powder. The surface modification of starch powder was achieved by curing SOMA mixed cassava starch powder in an oven at 120°C for 2 hours. The SOMA-g-STARCH was washed with acetone to remove excess SOMA. FTIR analysis of SOMA-g-STARCH was carried out and FTIR spectra are presented in Figure 3. As seen, SOMA-g-STARCH exhibits the absorption bands at around 1775 cm^−1^ and 1850 cm^−1^, corresponding the ester bonding formed by the ring opening relation between SOMA anhydride group and starch hydroxyl group. As a result, the starch powder surface becomes hydrophobic due to surface oil coverage. The starch hydroxyl groups are hindered, as indicated by the weak intensity of hydroxyl bands between 3000 and 3500 cm^−1^. Therefore, SOMA-g-STARCH exhibits hydrophobicity which is anticipated to exhibit good compatibility with PLA.

**Figure 4:**
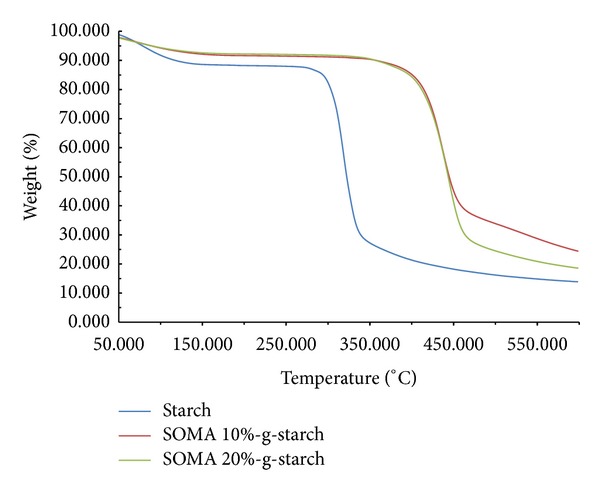
TGA thermograms of starch and soybean oil-g-cassava starch powder.

When considering TGA thermograms (Figure 4), it is found that the percent weight loss corresponding to bound moisture taken at the temperature of 125°C decreases in the following order: STARCH > SOMA10-g-STARCH > SOMA20-g-STARCH. These further confirm that the surface characteristic of SOMA-g-STARCH is hydrophobically modified by SOMA. 

### 3.3. PLA Filled with Cassava Starch-g-Soybean Oil Maleate

**Figure 5:**
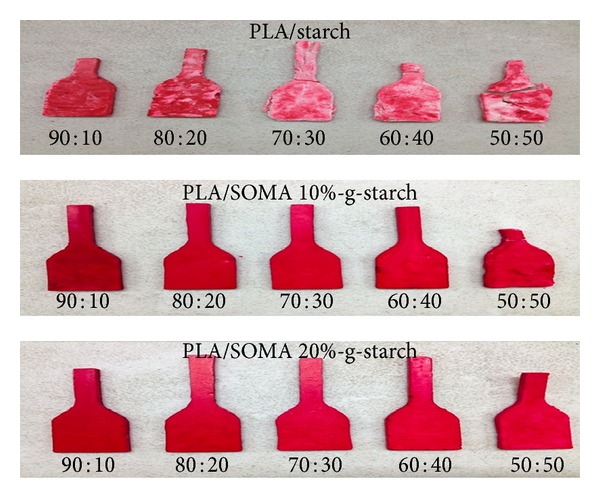
Shows photographs of PLA/SOMA-g-STARCH composites dyed with red disperse dye.

**Figure 6:**
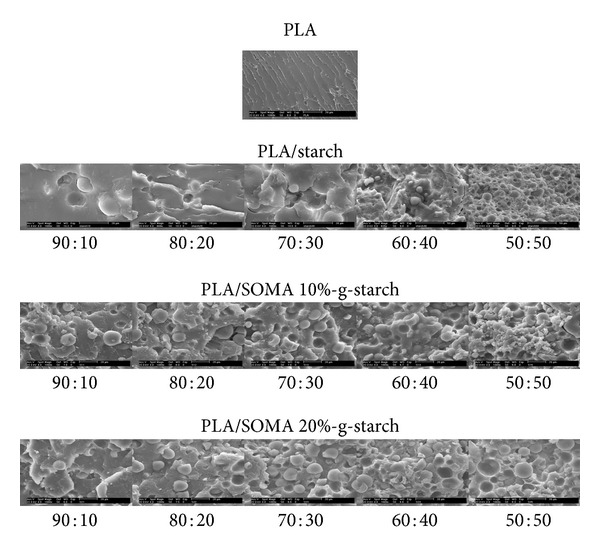
SEM images of PLA/Starch composites and PLA/SOMA-g-STARCH composites.

The PLA/SOMA-g-STARCH composites with various PLA : SOMA-g-STARCH weight ratios were prepared. Distribution of SOMA-g-STARCH particles in PLA matrix was observed by disperse dye coloration and SEM analysis.  Figure 5 shows photographs of PLA/SOMA-g-STARCH composites dyed with red disperse dye. PLA is dyeable with disperse dye. On the other hand, starch due to its hydrophilicity exhibits no affinity to disperse dye (hydrophobic dye). In this study, dye coloration is a very useful technique to observe the distribution of starch powder in PLA matrix. As seen, the white area which is indicative of starch agglomerate (starch exhibits no affinity to disperse dye) is clearly evident in case of PLA/STARCH composite. In contrast, SOMA-g-STARCH is distributed evenly, indicating good dispersion of SOMA-g-STARCH in PLA matrix. These results confirm the hypothesis that starch particle without surface hydrophobicity modification is poorly compatible with PLA. As a result of poor compatibility, PLA/STARCH composites are easy to be fragile as seen in Figure 5. Fracture analysis by SEM is shown in Figure 6. Starch particle distribution is observed. By surface modification, the starch powder morphology including size and shape remains unchanged. Interestingly, the particle distribution between unmodified starch and SOMA-g-STARCH is rather different. In case of PLA/Starch composites, starch agglomerates are dominant, leaving bulk area of PLA unfilled. Scattered particles are observed. At the agglomeration area (50 : 50 PLA : Starch), starch particles are densely packed and loosely attached to PLA matrix. For PLA/SOMA-g-STARCH composites, the results show that SOMA-g-STARCH particles are well dispersed in PLA matrix as a result of compatibility among two phases. However, adhesion of SOMA-g-STARCH particles is weak, as observed by the detachment of filler particles from PLA matrix.

### 3.4. Impact Strength and Tensile Properties

**Figure 7:**
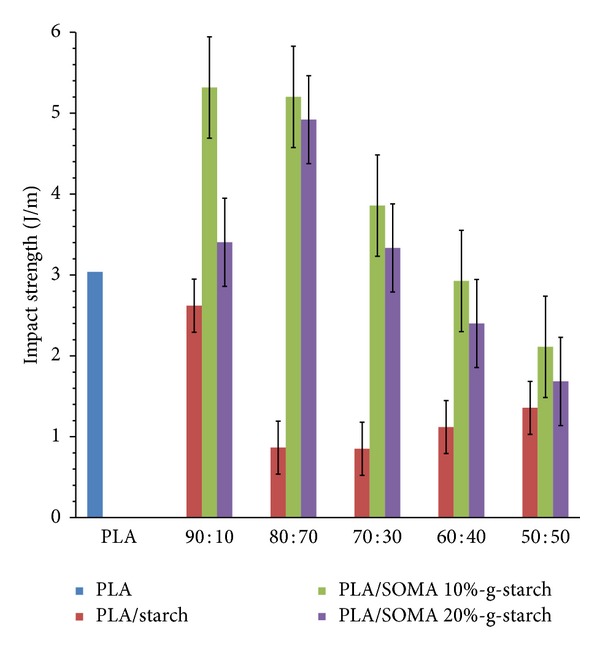
Impact strength values of PLA, PLA/Starch composites, and PLA/SOMA-g-STARCH composites.

PLA is considered as the brittle polymer derived from low thermal stability occurring during heat processing, leading to its application restriction. Therefore, an improvement of PLA impact strength is one of the challenging research topics. In this study, an effect of SOMA-g-STARCH on impact strength was evaluated and the results are shown in Figure 7. As seen, the impact strength of pristine PLA is about 3 J/m which is very low, resulting from the rigid nature of PLA. The loading of unmodified starch into PLA tends to reduce the impact strength due to incompatibility between starch phase and PLA matrix. In contrast, the loading of SOMA-g-STARCH into PLA results in a significant improvement of impact strength. From Figure 7, in case of SOMA-g-STARCH contents below 30 wt%, the impact strength values of composites are found to be higher than PLA and PLA/starch composites due to better interfacial adhesion and good particles distribution. However, PLA/SOMA-g-STARCH composites with SOMA-g-STARCH content higher than 30 wt% exhibit a decrease in the impact strength, arising from the problem of particle agglomeration. It is thought that soybean oil on the particle surface may play a role in improving the impact strength deriving from its plasticity-like behavior. 

**Figure 8:**
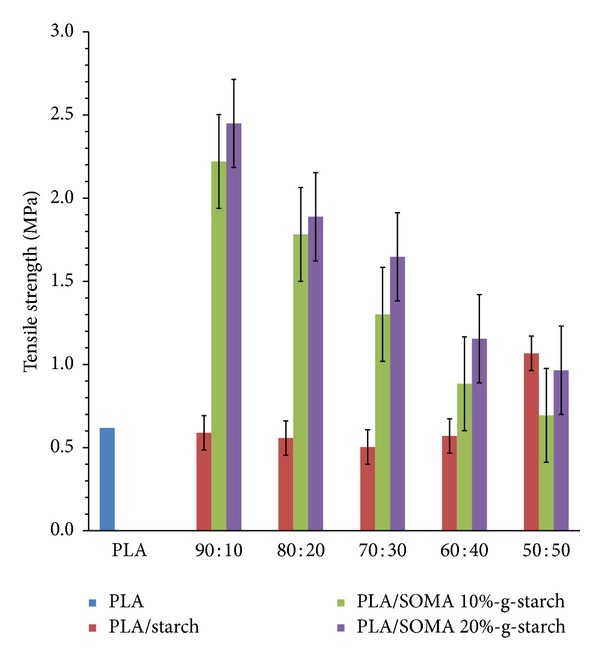
Tensile strength values of PLA, PLA/Starch composites, and PLA/SOMA-g-STARCH composites.

**Figure 9:**
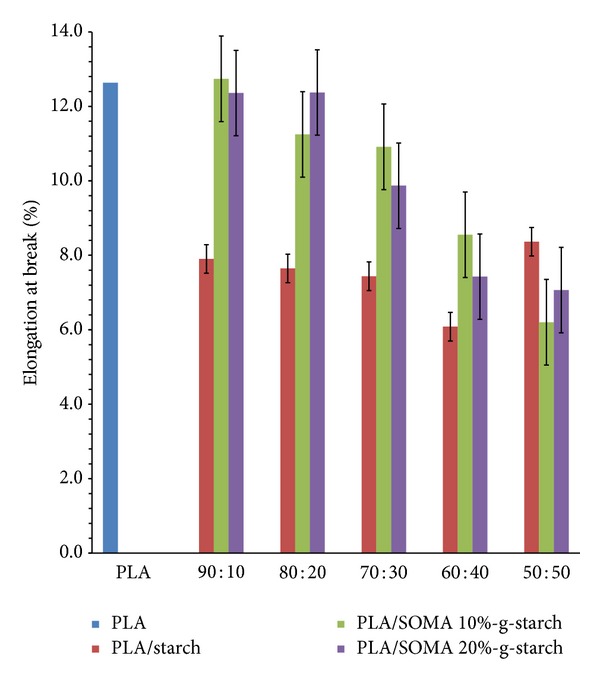
Percent elongation at break values of PLA, PLA/Starch composites, and PLA/SOMA-g-STARCH composites.

Tensile strength and percent elongation at break of PLA/SOMA-g-STARCH composites are shown in Figures 8 and 9, respectively. The tensile strength of PLA that underwent melt extrusion is only 0.6 MPa which is unusually low when compared to the other work, showing that PLA exhibited the tensile strength in the range of 40–50 MPa. This is due to the reason that PLA was substantially depolymerized by melt processing, resulting in a significant reduction of PLA molecular weight. Moreover, tensile properties were measured on twice melt PLA samples. Therefore, it should be noted that those obtained mechanical properties values in this experiment are discussed in relative value to each other. The addition of unmodified starch leads to a noticeable reduction in tensile strength due to phase separation between PLA matrix and starch particles, arising from poor interfacial adhesion. The improvement of interfacial adhesion between starch powder and PLA was achieved by surface modification of starch with soybean oil maleate. As a result, the tensile strength values of PLA/SOMA-g-STARCH composites are higher than those of PLA/starch composites. In a similar manner, the percent elongations of PLA/starch composites are inferior to that of pristine PLA due to imperfect mixing of PLA and starch. The boundary crack is responsible for the poor tensile property of PLA/starch composites. A relative decrease in the percent elongations of PLA/SOMA-g-STARCH composites is observed with an increase in SOMA-g-STARCH content, reflecting that phase separation is likely to occur when a high amount of SOMA-g-STARCH above 10 wt% is employed. 

## 4. Conclusions

Maleated soybean oil whose functional group and chemical structure were confirmed by FTIR and ^1^H NMR was successfully prepared by grafting reaction of soybean oil with maleic anhydride using dicumyl peroxide as an initiator. Then, maleated soybean oil was employed for surface modification of cassava starch powder, producing soybean oil grafted starch powder (SOMA-g-STARCH). FTIR analysis and TGA analysis confirmed that SOMA-g-STARCH surface changed from hydrophilicity to hydrophobicity. The incorporation of SOMA-g-STARCH into polylactic acid by melt extrusion mixing using PLA : SOMA-g-STARCH weight ratios of 90 : 10, 80 : 20, 70 : 30, 60 : 40, and 50 : 50 was carried out. It was found that the loading of SOMA-g-STARCH into PLA resulted in a significant improvement of impact strength, in case of SOMA-g-STARCH contents which have below 30 wt% due to better interfacial adhesion and good particles distribution. However, PLA/SOMA-g-STARCH composites with SOMA-g-STARCH content higher than 30 wt% exhibited a decrease in the impact strength, arising from the problem of particle agglomeration. In a similar manner, polymer composites of PLA filled with cassava starch-g-soybean oil maleate exhibited higher mechanical properties than polymer composites of PLA filled with unmodified starch powder. It was thought that soybean oil on the particle surface might play a key role in improving the compatibility as well as performing the plasticity-like behavior.
